# Factors Significantly Associated with Postoperative Neck Pain Deterioration after Surgery for Cervical Ossification of the Posterior Longitudinal Ligament: Study of a Cohort Using a Prospective Registry

**DOI:** 10.3390/jcm10215026

**Published:** 2021-10-28

**Authors:** Masao Koda, Toshitaka Yoshii, Satoru Egawa, Kenichiro Sakai, Kazuo Kusano, Yukihiro Nakagawa, Takashi Hirai, Kanichiro Wada, Keiichi Katsumi, Atsushi Kimura, Takeo Furuya, Satoshi Maki, Narihito Nagoshi, Kota Watanabe, Tsukasa Kanchiku, Yukitaka Nagamoto, Yasushi Oshima, Kei Ando, Hiroaki Nakashima, Masahiko Takahata, Kanji Mori, Hideaki Nakajima, Kazuma Murata, Shunji Matsunaga, Takashi Kaito, Kei Yamada, Sho Kobayashi, Satoshi Kato, Tetsuro Ohba, Satoshi Inami, Shunsuke Fujibayashi, Hiroyuki Katoh, Haruo Kanno, Hiroshi Takahashi, Kengo Fujii, Masayuki Miyagi, Gen Inoue, Masashi Takaso, Shiro Imagama, Yoshiharu Kawaguchi, Katsushi Takeshita, Masaya Nakamura, Morio Matsumoto, Atsushi Okawa, Masashi Yamazaki

**Affiliations:** 1 Department of Orthopedic Surgery, Faculty of Medicine, University of Tsukuba, 1-1-1 Tennodai, Tsukuba 305-8575, Japan; hhtaka@tsukuba-seikei.jp (H.T.); kengox15feb@gmail.com (K.F.); masashiy@md.tsukuba.ac.jp (M.Y.); 2 Department of Orthopedic Surgery, Tokyo Medical and Dental University, 1-5-45 Yushima, Bunkyo Ward, Tokyo 113-8519, Japan; yoshii.orth@tmd.ac.jp (T.Y.); egawa.orth@tmd.ac.jp (S.E.); hirai.orth@tmd.ac.jp (T.H.); okawa.orth@tmd.ac.jp (A.O.); 3 Department of Orthopedic Surgery, Saiseikai Kawaguchi General Hospital, 5-11-5 Nishikawaguchi, Kawaguchishi, Saitama 332-8558, Japan; kenitiro1122@gmail.com; 4 Department of Orthopedic Surgery, Kudanzaka Hospital, 1-6-12 Kudanminami, Chiyodaku, Tokyo 102-0074, Japan; kz_kusano@yahoo.co.jp; 5 Department of Orthopaedic Surgery, Wakayama Medical University Kihoku Hospital, 219 Myoji, Katsuragi-cho, Itogun, Wakayama 649-7113, Japan; yukihiro19670916@gmail.com; 6 Department of Orthopedic Surgery, Hirosaki University Graduate School of Medicine, 5 Zaifucho, Hirosaki, Aomori 036-8562, Japan; wadak39@hirosaki-u.ac.jp; 7 Department of Orthopedic Surgery, Niigata University Medicine and Dental General Hospital, 1-754 Asahimachidori, Chuo Ward, Niigata 951-8520, Japan; kkatsu_os@yahoo.co.jp; 8 Department of Orthopedics, Jichi Medical University, 3311-1 Yakushiji, Shimotsuke 329-0498, Japan; akimura@jichi.ac.jp (A.K.); dtstake@gmail.com (K.T.); 9 Department of Orthopedic Surgery, Chiba University Graduate School of Medicine, 1-8-1 Inohana, Chuo Ward, Chiba 260-0856, Japan; takeo251274@yahoo.co.jp (T.F.); satoshimaki@gmail.com (S.M.); 10 Department of Orthopaedic Surgery, School of Medicine, Keio University, 35 Shinanomachi, Shinjuku Ward, Tokyo 160-8582, Japan; nagoshiation@gmail.com (N.N.); watakota@gmail.com (K.W.); masa@a8.keio.jp (M.N.); morio@sc.itc.keio.ac.jp (M.M.); 11 Department of Orthopedic Surgery, Yamaguchi Rosai Hospital, 1315-4 Onoda, Onoda-City 756-0095, Japan; t-kanchi@pg8.so-net.ne.jp; 12 Department of Orthopedic Surgery, Osaka Rosai Hospital, 1179-3 Nagasonecho, Sakaishi 591-8025, Japan; 7gam0to@gmail.com; 13 Department of Orthopaedic Surgery, Faculty of Medicine, The University of Tokyo, 7-3-1 Hongo, Bunkyo-ku, Tokyo 113-0033, Japan; yoo-tky@g.ecc.u-tokyo.ac.jp; 14 Department of Orthopedic Surgery, Nagoya University Graduate School of Medicine, 65 Tsurumaicho, Showa Ward, Nagoya 466-8550, Japan; hirospine@med.nagoya-u.ac.jp (H.N.); keikeiando@hotmail.co.jp (K.A.); imagama@med.nagoya-u.ac.jp (S.I.); 15 Department of Orthopaedic Surgery, Faculty of Medicine and Graduate School of Medicine, Hokkaido University, Kita 15, Nishi 7, Sapporo 060-8638, Japan; takamasa@med.hokudai.ac.jp; 16 Department of Orthopaedic Surgery, Shiga University of Medical Science, Tsukinowa-cho, Seta, Otsu 520-2192, Japan; kanchi@belle.shiga-med.ac.jp; 17 Department of Orthopaedics and Rehabilitation Medicine, Faculty of Medical Sciences, University of Fukui, 23-3 Matsuoka Shimoaizuki, Eiheiji-cho, Yoshida-gun, Fukui 910-1193, Japan; nhideaki@u-fukui.ac.jp; 18 Department of Orthopedic Surgery, Tokyo Medical University, 6-7-1 Nishishinjuku, Shinjuku-ku, Tokyo 160-0023, Japan; kaz.mur26@gmail.com; 19 Department of Orthopedic Surgery, Kawamoto Memorial Clinic, 5397-3 Yoshinocho, Kagoshima-City 892-0871, Japan; shunji622@gmail.com; 20 Department of Orthopedic Surgery, Graduate School of Medicine, Osaka University, 2-2 Yamadaoka, Suita-shi, Osaka 565-0871, Japan; takashikaito@gmail.com; 21 Department of Orthopaedic Surgery, Kurume University School of Medicine, 67 Asahi-machi, Kurume-shi 830-0011, Japan; yamada_kei@med.kurume-u.ac.jp; 22 Department of Orthopedic Surgery, Hamamatsu University School of Medicine, 1-20-1 Handayama, Hamamatsu 431-3125, Japan; dr.shokobayashi@gmail.com; 23 Department of Orthopaedic Surgery, Graduate School of Medical Sciences, Kanazawa University, 13-1 Takara-machi, Kanazawa 920-8641, Japan; skato323@gmail.com; 24 Department of Orthopedic Surgery, University of Yamanashi, 1110 Shimokato, Chuo Ward, Yamanashi 409-3898, Japan; tooba@yamanashi.ac.jp; 25 Department of Orthopaedic Surgery, Dokkyo Medical University School of Medicine, 880 Kitakobayashi, Mibu-machi, Shimotsuga-gun, Tochigi 321-0293, Japan; iinami@dokkyomed.ac.jp; 26 Department of Orthopaedic Surgery, Graduate School of Medicine, Kyoto University, 54 Kawahara-cho, Shogoin, Sakyo-ku, Kyoto 606-8507, Japan; shfuji@kuhp.kyoto-u.ac.jp; 27 Department of Orthopedic Surgery, Surgical Science, Tokai University School of Medicine, 143 Shimokasuya, Isehara 259-1193, Japan; hero@tokai-u.jp; 28 Department of Orthopaedic Surgery, Tohoku University School of Medicine, 1-1 Seiryomachi, Aoba Ward, Sendai 980-8574, Japan; kanno-h@isis.ocn.ne.jp; 29 Department of Orthopaedic Surgery, Kitasato University School of Medicine, 1-15-1 Kitasato, Minami Ward, Sagamihara 252-0375, Japan; masayuki008@aol.com (M.M.); ginoue@kitasato-u.ac.jp (G.I.); mtakaso@kitasato-u.ac.jp (M.T.); 30 Department of Orthopedic Surgery, Faculty of Medicine, University of Toyama, 2630 Sugitani, Toyama 930-0194, Japan; zenji@med.u-toyama.ac.jp

**Keywords:** ossification of the posterior longitudinal ligament, cervical spine, surgery, neck pain

## Abstract

Postoperative neck pain has been reported as an unsolved postoperative complication of surgery for cervical ossification of the posterior longitudinal ligament (OPLL). The aim of the present study was to elucidate factors having a significant association with postoperative deterioration of neck pain in cervical OPLL patients. We studied a cohort of patients in a prospective registry of 478 patients who had undergone cervical spine surgery for cervical OPLL. We excluded those without evaluation of preoperative neck pain. Therefore, 438 patients were included in the present study. Neck pain was evaluated with the visual analogue scale (VAS, 0–100 mm). Postoperative neck pain deterioration was defined as a ≥20 mm increase of VAS neck pain. Patient factors, neurological status, imaging factors and surgical factors were assessed. Univariate analyses followed by multivariate analysis using stepwise logistic regression was performed. Six months after surgery, 50 (11.6%) patients showed postoperative neck pain deterioration and 76 (17.4%) patients showed postoperative neck pain deterioration 2 years after surgery. Six months after surgery, the rate of neck pain deterioration was significantly higher in patients who had undergone posterior surgery. Two years after surgery, the number of levels fused was significantly correlated with neck pain deterioration.

## 1. Introduction

Ossification of the posterior longitudinal ligament (OPLL), widely observed in Asian people, is ectopic ligamentous ossification that can cause spinal cord or nerve root compression, or both, when the ossification foci thicken [1,2,3]. For patients with impairment of activities of daily living caused by OPLL, decompression-fusion surgery is recommended [4]. Although recent progress in understanding the pathology and development of spinal instrumentation could produce a better neurological outcome, postoperative neck pain has been reported as an unsolved postoperative complication of surgery for cervical OPLL [5,6]. Our ultimate goal is to attenuate postoperative neck pain after decompression-fusion surgeries for cervical OPLL. As a first step, the primary aim of the present study was to elucidate factors having a significant association with postoperative deterioration of neck pain in a cohort of patients, using a prospective multicenter registry of patients with surgically-treated cervical OPLL.

## 2. Materials and Methods 

**Figure 1:**
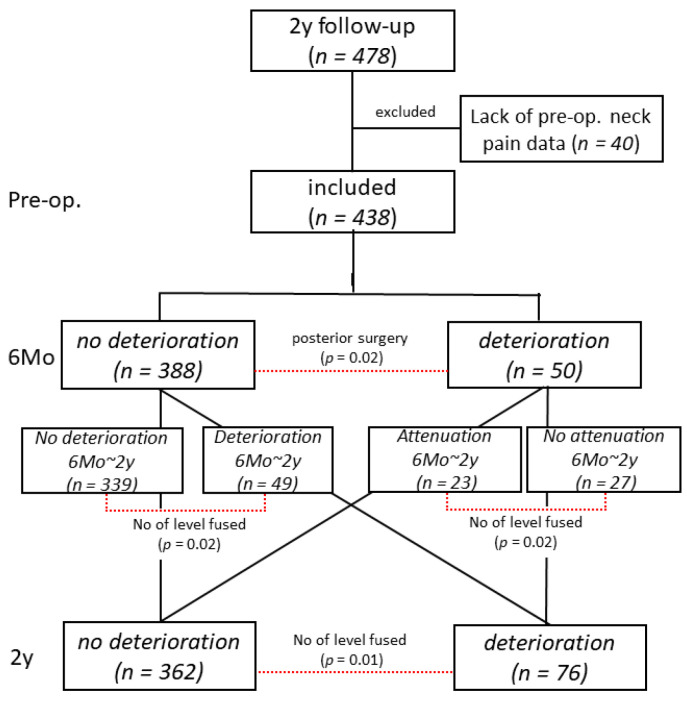
Patients who had undergone cervical spine surgery for myelopathy caused by cervical OPLL. We excluded those without evaluation of preoperative neck pain. We ultimately included 438 patients with treated cervical OPLL.

We studied a cohort of patients in a prospective registry of 478 patients who had undergone cervical spine surgery for myelopathy caused by cervical OPLL. Among those patients, we excluded those without evaluation of preoperative neck pain. We ultimately included 438 patients with treated cervical OPLL (Figure 1, Table 1). Written informed consent was obtained from all the participants. 

Surgical procedures included in the present registry were as follows: Laminoplasty includes both open-door laminoplasty and French-door laminoplasty. Struts to keep laminae enlarged includes plate, a hydroxyapatite spacer and an autologous spinous process were used. Posterior decompression and fusion means posterior instrumented fusion combined with laminoplasty/laminectomy. Anterior decompression and fusion consists of corpectomy and floating/extirpation of ossified lesions and bone grafts augmented with a plate. A-P means anterior decompression and posterior instrumented fusion. Indication for surgical treatment was based on myelopathy causing impairment of activities of daily living. Precise surgical indication, choice of surgical procedures and detailed surgical procedures are different at each institution. Surgeons belonging to each institution performed surgeries; therefore, 72 surgeons performed surgery in the present registry.

Neck pain was evaluated with the visual analogue scale (VAS, 0–100 mm) preoperatively, then 6 months and 2 years after surgery. Postoperative neck pain deterioration was defined as a ≥20 mm increase of VAS neck pain. Patient factors, including age at surgery, sex, body mass index, disease duration and diabetes mellitus, were assessed. Neurological status was assessed with the Japanese Orthopedic Association score for evaluating cervical myelopathy (JOA score; 0–17 points [7]) preoperatively, and at 6 months and 2 years after surgery. JOA score is widely known as a well standardized evaluation/classification tool for cervical myelopathy, and its consistency/reproducibility between surgeons is also well known. The recovery rate of JOA score (%) was calculated as follows: (postoperative JOA score—preoperative JOA score)/(17 (full score)—preoperative JOA score) × 100 [8]. Imaging factors were analyzed preoperatively, and at 6 months and 2 years after surgery, as follows: type of OPLL (continuous, segmental, mixed, and localized types [9]), canal narrowing rate (thickness of OPLL at its peak level/anteroposterior diameter of corresponding spinal level (%)), postoperative change of C2-7 angle (angle between inferior endplates of C2 and C7 vertebral bodies), change of C2-7 range of motion (ROM; subtraction of C2-7 angle from extension position to flexion position) and spinal cord signal intensity change in magnetic resonance imaging (MRI) T2-weighted images. Surgical factors, including surgical procedures (laminoplasty, posterior decompression with instrumented fusion (PDF), anterior decompression and fusion (ADF), anteroposterior decompression and fusion (A-P)), and number of levels fused, were determined. 

We first performed univariate analyses followed by multivariate analysis using stepwise logistic regression to elucidate the independent factors having a significant positive association with postoperative neck pain deterioration. Postoperative neck pain deterioration, which was defined as a ≥20 mm increase of VAS neck pain, was set as a response variable. The abovementioned factors, including background factors for the patients, surgical factors, neurological status, and imaging factors, were set as explanatory variables. All the factors were checked for their multicollinearity with each other before univariate analyses. Factors with *p* < 0.1 in initial univariate analyses were then analyzed by stepwise logistic regression. Factors with *p* < 0.05 were determined as independent factors having a significant positive association with postoperative neck pain deterioration. Odds ratio and 95% confidence interval were calculated for factors screened. In addition, we performed statistical analyses for factors having a significant association with neck pain deterioration or attenuation between 6 months and 2 years after surgery. Comparisons between patients in the group not showing neck pain deterioration 6 months after surgery demonstrated both pain deterioration and no deterioration 2 years after surgery. In other words, this group showed neck pain deterioration between 6 months and 2 years after surgery. By contrast, comparisons between patients in the group showing neck pain deterioration 6 months after surgery demonstrated both pain deterioration and no deterioration 2 years after surgery. In other words, this group showed neck pain attenuation between 6 months and 2 years after surgery (Figure 1). All the statistical analyses were conducted with JMP statistical analytics software (version 12.0; SAS Institute, Cary, NC, USA) under the supervision of a biostatistician. Those statistical analyses were performed on data obtained 6 months and 2 years after surgery.

## 3. Results

Six months after surgery, neck pain significantly decreased from preoperative VAS (41.6 ± 31.6 mm) to 36.6 ± 29.1 mm (*p* = 0.04, Tukey Kramer HSD test). Neck pain 2 years after surgery (38.5 ± 30.7 mm) did not show a significant change compared with preoperative neck pain (*p* = 0.39). Fifty (11.6%) patients showed postoperative neck pain deterioration 6 months after surgery, whereas the remaining 438 (88.4%) patients showed no deterioration and 76 (17.4%) patients showed postoperative neck pain deterioration 2 years after surgery (Table 2). The estimated sample size was 593 cases to obtain enough power (=0.8) calculated with power analysis (α error: 0.05, overall power: 0.58, power analysis with JMP ver. 12) and effect size was 0.67. 

Among 50 patients showing postoperative neck pain deterioration 6 months after surgery, 23 showed attenuation of neck pain, and the remaining 27 showed no recovery from neck pain. Thus, neck pain deterioration in 49 of 76 patients occurred between 6 months and 2 years after surgery. Six months after surgery, the rate of neck pain deterioration was significantly higher in patients who had undergone laminoplasty or PDF than in those who had undergone ADF or A-P (*p* = 0.02, Table 3). Two years after surgery, the number of levels fused was significantly correlated with neck pain deterioration (*p* < 0.01, Table 3). By initial univariate analyses, the number of levels fused was the only screened factor; therefore, we no longer performed multivariate analysis 2 years after surgery. Number of levels fused was associated with neck pain deterioration between 6 months and 2 years after surgery (*p* = 0.02, Figure 1). Number of levels fused was negatively associated with neck pain attenuation from 6 months to 2 years after surgery (*p* = 0.02, Figure 1). The other factors, including patient factors (preoperative VAS neck pain, BMI, diabetes, etc.) and imaging factors, had no significant influence on neck pain deterioration. The cut-off value of the “No. of levels fused” to have a significant association with neck pain deterioration 2 years after surgery was six levels (ROC analysis with JMP ver. 12, AUC = 0.67). Therefore, fusion surgery for six levels or more could cause neck pain deterioration.

## 4. Discussion

The present results showed that a posterior approach was significantly associated with postoperative neck pain deterioration 6 months after surgery. In addition, there was no significant difference in the proportion of those with neck pain deterioration between patients who had undergone PDF or LMP. This suggests that the surgical invasion to posterior paraspinal muscles might cause postoperative neck pain. Previous reports revealed a significant association between muscle invasion and postoperative neck pain, specifically axial pain [10,11]. Hosono reported that avoiding surgical detachment of C7 spinous process nuchal ligament insertion and C2 semispinalis or paraspinal muscles attenuated postoperative axial neck and shoulder pain [12]. Ishibashi reported that the preservation of C2 muscle attachment by C3 laminectomy (not laminoplasty) can attenuate postoperative neck pain [13]. These lines of evidence suggest a close relationship between muscle invasion and postoperative neck pain. The present results for neck pain deterioration 6 months after surgery are consistent with those described previously. Efforts to decrease surgical invasion to paraspinal muscles related to a posterior approach are essential to attenuate neck pain deterioration 6 months after surgery.

Two years after surgery there was a significant association between neck pain deterioration and the number of levels fused, but not surgical procedures. This suggests that there is no significant influence of a posterior approach-related paraspinal muscle injury to neck pain deterioration in the mid-to-long term. By contrast, the number of levels fused was significantly associated with postoperative neck pain deterioration. We found that the neck pain in 49 of 388 patients without neck pain deterioration 6 months after surgery worsened between 6 months and 2 years after surgery. In addition, the neck pain in 23 of 50 patients with neck pain deterioration 6 months after surgery reduced between 6 months and 2 years after surgery. The number of levels fused was significantly associated with this late neck pain deterioration, whereas the type of surgical procedure was not significantly associated with this late neck pain deterioration. These lines of evidence suggest that the number of levels fused might be independent of posterior surgery-related muscle damage. The significant association between number of levels fused and neck pain deterioration suggests that limited mobility of the cervical spine might cause neck pain. Previous reports indicated that excessive longer external fixation using a neck collar can cause greater neck pain than a shorter time of external fixation after cervical spine surgery [14]. Moreover, a recent meta-analysis comparing cervical disc arthroplasty and anterior cervical diskectomy and fusion found that neck pain was significantly lower in a group with disc arthroplasty than in a group with fusion [15,16,17,18]. These reports suggest a possible relationship between the restriction of cervical spine motion and neck pain deterioration. Paraspinal muscle atrophy induced by multilevel fusion surgery is a possible cause of motion restriction-related neck pain. Unfortunately, we collected MRI data only in sagittal images to evaluate spinal cord intensity change, resulting in a lack of axial MRI images which would enable us to assess paravertebral muscular atrophy.

A major limitation of the present study is that the present registry lacks sagittal alignment parameters, although recent studies revealed the close relationship between cervical sagittal alignment impairment and neck pain deterioration. Posterior surgery, even in fusion surgery, can lead to sagittal alignment worsening after surgery. Therefore, the assessment of sagittal alignment will be mandatory for elucidating the precise etiology of neck pain deterioration. Thus, a collection of sagittal alignment parameters should be considered in the near future. Another possible limitation is the lack of detailed pain data about its characteristics, precise location and relationship to motion, and so forth.

The precise etiology of neck pain deterioration after surgery remains to be fully elucidated; however, the present results suggest that the number of fusion levels must be kept at the minimum necessary to avoid postoperative neck pain after surgery for OPLL.

## 5. Conclusions

A posterior approach was significantly associated with neck pain deterioration 6 months after surgery for cervical OPLL, and the number of levels fused was significantly associated with neck pain deterioration 2 years after the surgery.

## Figures and Tables

**Table 1:** Patient demographics.

Demographics (*n* = 438)	
Male:Female (cases)	325:113
age at surgery (years old)	63.6 ± 11.6
disease duration (months)	42.8 (0–548)
body mass index	25.7 ± 4.3
diabetes (No. of cases)	134/438
JOA score (pts.)	
Pre-op.	10.5 ± 3.0
Post-op. 2y	13.9 ± 2.9
recovery rate (%)	46.2 ± 33.5
pre-op. neck pain (VAS, mm)	1.5 ± 31.6
Surgical procedures (cases)	
Laminoplasty	240 (C7 involvement: 154/240)
Posterior decompression & fusion	104
Anterior decompression & fusion	82
A-P	12
Number of levels fused	4.2 (1–8)
Imaging findings	
Type of OPLL (cases)	
Continuous	58
Segmental	161
Mixed	190
Localized	29
Canal narrowing rate (%)	43.9 ± 15.6
C2-7 angle (°)	9.3 ± 11.7 (ΔC2–7 angle: −1.1 ± 10.1)
range of motion (°)	26.7 ± 13.9 (ΔROM: −10.1 ± 15.6)
T2 high signal change (cases)	373/438

**Table 2:** Changes in VAS neck pain.

Neck Pain (VAS, 0–100 mm)	*p*-Value (vs. Pre-Op.)
pre-op.	41.6 ± 31.6 mm
post-op. 6Mo	36.6 ± 29.1 mm * 0.02
pain deterioration > 20 mm (cases)	50/438 (11.4%)
post-op. 2y	38.5 ± 30.7 mm 0.14
pain deterioration > 20 mm (cases)	76/438 (17.4%)

*: *p* < 0.05 vs. pre-op.

**Table 3:** Possible factors associated with postoperative neck pain deterioration.

Univariate Analyses	6 Mo	2 y
Patient factor		
age	0.80	0.36
sex	0.98	0.12
BMI	0.81	0.43
disease duration	0.28	0.38
DM	0.13	0.54
Neurological status		
JOA score recovery rate	0.02 *	0.20
Imaging factor		
types of OPLL	0.65	0.27
canal occupying ratio	0.25	0.67
ΔC2-7 angle	0.76	0.88
ΔC2-7 ROM	0.31	0.72
MRI T2 high signal	0.78	0.50
Surgical factors		
Surgical procedures	0.04 *	0.81
No. of levels fused	0.03 *	0.002 *
Multivariate analysis (6 Mo)		
JOA score recovery rate	0.20	
Surgical procedures	0.02 *	
No. of levels fused	0.40	

*: *p* < 0.05.

## Data Availability

The datasets generated during and analyzed during the current study are available from the corresponding author on reasonable request. The data are not publicly available due to privacy.
